# The neurological and non-neurological roles of the primary microcephaly-associated protein ASPM

**DOI:** 10.3389/fnins.2023.1242448

**Published:** 2023-08-03

**Authors:** Xingxuan Wu, Zheng Li, Zhao-Qi Wang, Xingzhi Xu

**Affiliations:** ^1^Guangdong Key Laboratory for Genome Stability and Disease Prevention and Marshall Laboratory of Biomedical Engineering, Shenzhen University Medical School, Shenzhen, Guangdong, China; ^2^Shenzhen University-Friedrich Schiller Universität Jena Joint PhD Program in Biomedical Sciences, Shenzhen University School of Medicine, Shenzhen, Guangdong, China; ^3^Laboratory of Genome Stability, Leibniz Institute on Aging-Fritz Lipmann Institute, Jena, Germany

**Keywords:** microcephaly, small brain, ASPM, MCPH5, neurogenesis, cancer

## Abstract

Primary microcephaly (MCPH), is a neurological disorder characterized by small brain size that results in numerous developmental problems, including intellectual disability, motor and speech delays, and seizures. Hitherto, over 30 MCPH causing genes (*MCPHs*) have been identified. Among these *MCPHs*, *MCPH5*, which encodes abnormal spindle-like microcephaly-associated protein (ASPM), is the most frequently mutated gene. ASPM regulates mitotic events, cell proliferation, replication stress response, DNA repair, and tumorigenesis. Moreover, using a data mining approach, we have confirmed that high levels of expression of ASPM correlate with poor prognosis in several types of tumors. Here, we summarize the neurological and non-neurological functions of ASPM and provide insight into its implications for the diagnosis and treatment of MCPH and cancer.

## 1. Introduction

Primary microcephaly (MCPH) is a neurodevelopmental disorder characterized by small brain size primarily due to the reduced cerebral cortex, varying degrees of intellectual disability (Woods et al., 2005; Mahmood et al., 2011; Phan and Holland, 2021; Zaqout and Kaindl, 2021; Gupta, 2023), and several additional neurological problems, such as seizures and epilepsy (Shen et al., 2005), with a prevalence ranging from 1/30,000 to 1/250,000. The development of brain relies on neurogenesis, the process by which neural stem cells proliferate, migrate, and differentiate to form neurons, is fundamental to normal brain development (Stiles and Jernigan, 2010; Isaev et al., 2019; Zhou et al., 2020). Neuron formation begins during embryogenesis and continues throughout life. In mammals, the size of the cerebral cortex is determined by the number of neurons it contains (Borrell and Calegari, 2014). In general, the human adult comprises about 86 billion neurons (Herculano-Houzel, 2012) and brain size range from 975 to 1,499 cm^3^. Studies have shown that a reduced number of neurons results in primary microcephaly, which is diagnosed when the occipital frontal circumference is smaller than two standard deviations below the mean at birth and/or smaller than three standard deviations below the mean after 1 year of age (Duerinckx et al., 2020).

At least 30 *MCPHs* (*MCPH1–MCPH30*) have been mapped to date. Mutations in *MCPH5*, which encodes the ASPM protein, are the most common cause of MCPH, accounting for around 40% of the patient population (Nicholas et al., 2009). To date, functions in cell division (Fish et al., 2006; Capecchi and Pozner, 2015), neurogenesis (Fujimori et al., 2014; Passemard et al., 2016), genome stability (Fujimori et al., 2008; Xu et al., 2021; Wu et al., 2022), and disease development (Fujimori et al., 2014; Liu et al., 2018) have all been annotated for ASPM. Here, we summarize the neurological and non-neurological functions of ASPM and provide insight into its implications for the diagnosis and treatment of MCPH and cancer.

## 2. Molecular and cellular characteristics of ASPM

**Figure 1:**
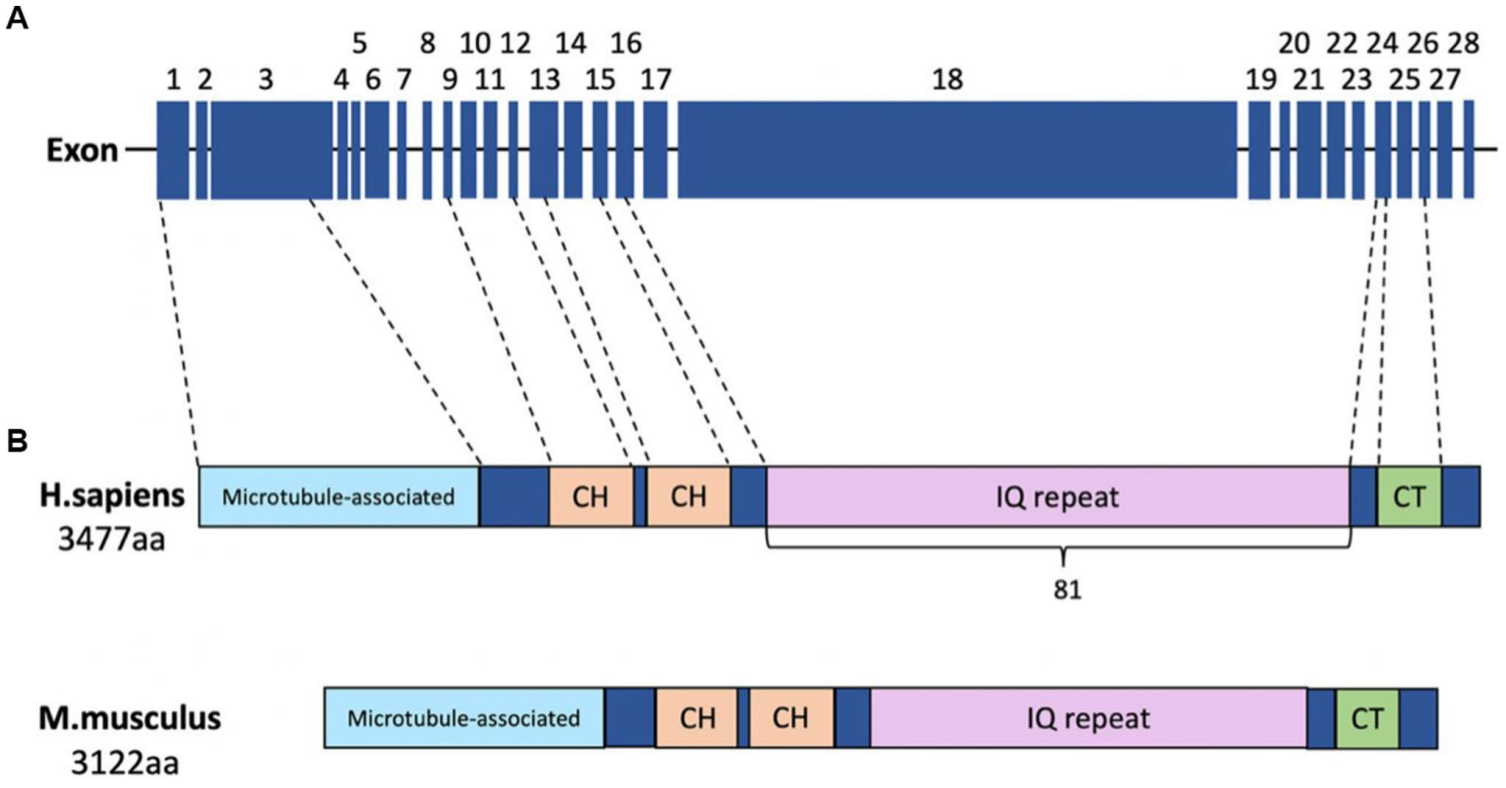
Schematic of human ASPM. **(A)** The 28 exons of the human *ASPM* gene, including the largest exon, exon 18 (4.7 kb). **(B)** Schematic showing the known domains of the human ASPM protein: N-terminal microtubule-binding domain (blue); two calponin homology (CH) domains (orange); 81 isoleucine and glutamine (IQ) repeats (pink); and the C-terminal (CT) domain (green).

To better understand ASPM functions in health and disease, it is important to delineate the structure and cellular roles of ASPM. As a member of the ASH (ASPM, SPD-2, and Hydin) domain-containing protein family, ASPM is the human homolog of the *Drosophila melanogaster* abnormal spindle protein (asp). ASPM is encoded by *MCPH5* on chromosome 1q31.3, a gene that was originally identified in studies of consanguineous Northern Pakistani families (Jamieson et al., 2000; Pattison et al., 2000; Bond et al., 2002). *MCPH5* has 28 exons and at least two alternative splicing isoforms: isoform 1 (full-length, amino acids 1–3,477) and isoform 2 (lacking the largest exon, exon 18, which encodes amino acids 1,356–2,940). Human ASPM protein contains four domains: a microtubule-binding domain (MTBD) at the N-terminal (NT), two calponin homology domains (CH), an isoleucine and glutamine domain (IQ motif), and a species-conserved C-terminal (CT; Figure 1).

The MTBD facilitates the localization of ASPM to the spindle pole and mediates an interaction between ASPM and microtubules that is responsible for the dynamic regulation of microtubules during cell division and neurogenesis (Jiang et al., 2017). The CH domains, commonly found in actin-binding proteins, are also thought to be involved in the interactions between ASPM and the actin cytoskeleton or microtubules (van der Voet et al., 2009). The 81 IQ repeats, many of which are organized into a higher-order repeat structure (Kouprina et al., 2005), are implicated in calmodulin binding (van der Voet et al., 2009).

**Figure 2:**
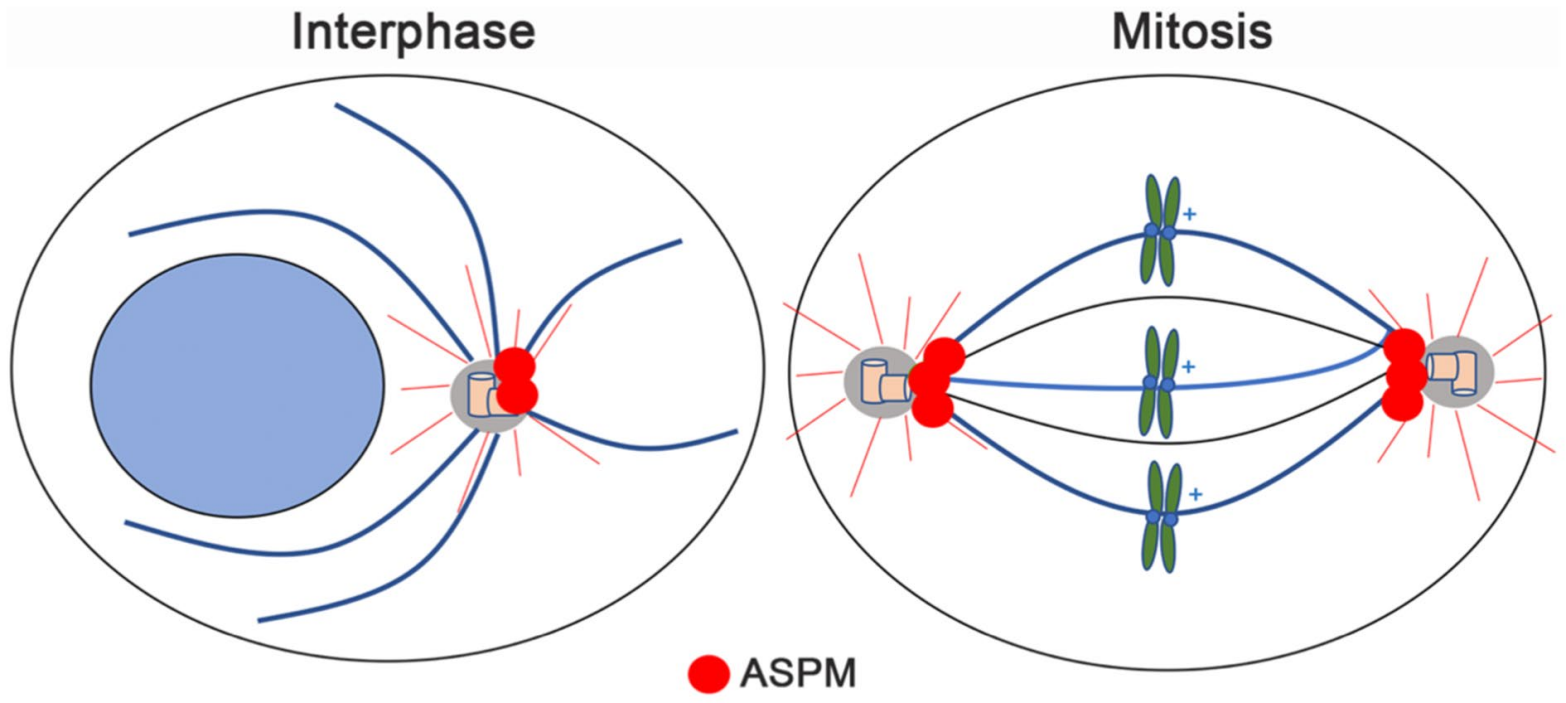
Schematic showing the cellular localization of ASPM during interphase and mitosis. ASPM signal (red dots) is primarily detected at the centrosome in interphase, while during cell division, ASPM signal is enriched at the spindle poles.

ASPM primarily localizes at the centrosome and the spindle poles during cell division (Tungadi et al., 2017; Sepulveda et al., 2018). It is predicted to be associated with cilia (Schou et al., 2014; Verdier et al., 2016). Studies in U2OS cells have shown expression of ASPM in the nucleus of interphase cells prior to nuclear envelope breakdown (Higgins et al., 2010). During mitosis, ASPM is recruited to the pericentriolar matrix surrounding γ-tubulin at the spindle pole in a microtubule-dependent manner (Kouprina et al., 2005; Higgins et al., 2010; Figure 2). Further studies have shown ASPM localization at the mitotic spindle poles (Kouprina et al., 2005; Bond and Woods, 2006; Fish et al., 2006) and the midbody ring (Paramasivam et al., 2007) in mammals (Bond and Woods, 2006) and rat neuronal progenitors in the embryonic neocortex (Paramasivam et al., 2007).

ASPM localization at the centrosome and spindle poles, is primarily known for regulating symmetric cell division, during which a mother cell divides into two identical daughter cells (Kouprina et al., 2005; Neumuller and Knoblich, 2009). The correct orientation of the spindle apparatus is a key determinant of symmetric cell division that ensures the accurate segregation of chromosomes (Fish et al., 2006; Higgins et al., 2010; Gai et al., 2016). In neural stem cells and progenitor cells, symmetric division is essential for the expansion of the progenitor cell pool and the generation of an adequate number of neurons during brain development (Fish et al., 2008; Knoblich, 2008; Neumuller and Knoblich, 2009; Jayaraman et al., 2018). Meanwhile, maintaining a balance of symmetric and asymmetric cell division is critical for normal brain development and tissue homeostasis (Gomez-Lopez et al., 2014; Taverna et al., 2014).

ASPM interacts with the minus ends of microtubules and plays a vital role in spindle assembly and orientation, microtubule-based transport, and cytokinesis (Higgins et al., 2010; Tungadi et al., 2017). In mouse embryonic neuroepithelial cells, loss of ASPM altered neuroepithelial cleavage plane orientation, resulting in deviation of the spindle position and an increase in asymmetric division rather than symmetric division (Fish et al., 2006). In the developing cerebellum, knockout of ASPM in cerebellar granule neuron progenitor cells impaired mitotic progression and altered division pattern orientation (Williams et al., 2015). Interestingly, two truncated forms of ASPM (missing exons 1–7 or 1717 C-terminal amino acids) in mice caused microcephaly, while only the mice lacking the C-terminal domain showed spindle misorientation (Pulvers et al., 2010; Capecchi and Pozner, 2015).

Mechanisms underlying ASPM’s regulation of spindle orientation have been investigated in several studies. For instance, Gai et al. identified an interaction between the cytokinesis regulator citron kinase (CITK), also known as *MCPH17*, and ASPM. CITK served as a downstream factor of ASPM, modulating spindle orientation in a kinase-dependent manner (Gai et al., 2016), and its localization at the spindle poles was ASPM-dependent. Moreover, overexpression of CITK in ASPM-depleted HeLa cells rescued the misorientation phenotype, demonstrating that these two microcephaly proteins function together to regulate spindle orientation (Gai et al., 2016). In another study, Jiang et al. used X-ray crystallography to identify a complex (ASPM-p60/p80) comprising of ASPM and the p60/p80 subunits of katanin, a microtubule-severing ATPase (Jiang et al., 2017). In *Drosophila*, ASPM-dependent recruitment of katanin to the microtubules enhanced the minus-end blocking activity of ASPM, which could suppress microtubule minus-end growth, while disruption of the interaction between ASPM and katanin caused impaired spindle orientation and poleward flux (Schoborg et al., 2015). In addition, T Schoborg et al. denmostrated that *Asp-*CaM (*Drosophila melanogaster* calmodulin) complex is required for centrosome-pole cohesion and centrosome inheritance in neural stem cells (Schoborg et al., 2015). These studies revealed the role of ASPM, together with its binding partners, in regulating symmetric cell division by modulating microtubule dynamics and spindle orientation.

## 3. ASPM in neurogenesis

Neurogenesis is a highly intricate and precise process involving the generation of functional neurons from neural progenitor cells (NPCs; Ming and Song, 2005, 2011). In mammals, two major brain regions are responsible for neurogenesis: the ventricular zone (VZ) and the subventricular zone (SVZ). During the early stage of neocortex development, the embryonic telencephalon wall is formed, consisting of neuroepithelial cells with apicobasal polarity (Woodworth et al., 2012; Jayaraman et al., 2018). These neuroepithelial cells undergo dynamic nuclear migration along the apical-basal axis in coordination with the cell cycle (Fish et al., 2008). Subsequently, they differentiate into multipotent NPCs capable of generating various cell types, including neurons and glial cells. Radial glial cells, which are located in the VZ, generate neurons and maintain self-renewal through multiple rounds of asymmetric divisions (Misson et al., 1988; Noctor et al., 2001). Additionally, radial glial cells can generate intermediate progenitors, which translocate to the SVZ and undergo symmetric proliferation or neurogenic divisions (Noctor et al., 2001; Jayaraman et al., 2018; de Almeida et al., 2023).

### 3.1. ASPM expression during neurogenesis

In mice, ASPM exhibits high expression levels in the cerebral cortical VZ at embryonic day (E) 14.5, when there are many progenitor cells. Its levels begin to decrease at E16.5 and are greatly reduced by postnatal day (P) 0, when the cortical VZ is fully formed (Bond et al., 2002). After birth, ASPM is also continuously expressed in zones of postnatal neurogenesis and adult tissues such as the dentate gyrus, cerebellar granule neurons, and the SVZ of the rostral migratory stream fated to become olfactory bulb neurons (Bond et al., 2002; Kouprina et al., 2005; Marinaro et al., 2011; Fujimori et al., 2014; Williams et al., 2015). These observations suggest that ASPM is preferentially expressed during cerebral cortical neurogenesis both before and after birth (Bond et al., 2002). Furthermore, the centrosomal localization of ASPM during interphase and mitotic spindle localization during mitosis has been demonstrated in mouse neuroepithelial cells at E12.5 (Kouprina et al., 2005).

The determinats of the cerebral cortex size include neurons number and neuronal migration during neurogenesis. In general, the final number of cortical neurons have a fundamental impact on the size of the mature cerebral cortex (Fernandez et al., 2016). Besides, B Nadarajah. et al. showed the importance of early-generated neurons in the layer formation and cortical connection establishment and elucidatied migration of neurons occurs during the whole period of corticogenesis and along multiple tangential routes to their destinations in the developing cortex, newly generated neurons must migrate to their appropriate locations within the developing brain to integrate into the cortical layers. Indicating the role of neuronal migration in maintaining cortical surface area (Nadarajah and Parnavelas, 2002; Nadarajah et al., 2003). Despite ASPM’s high expression during neurogenesis, its specific function in this context remains poorly understood. Several studies have shown that ASPM expression is required to balance symmetric proliferative division and differentiation in NPCs (Kouprina et al., 2005; Zhong et al., 2005; Fish et al., 2006; Horvath et al., 2006; Fujimori et al., 2014), as well as for neuronal migration (Buchman et al., 2011; Fujimori et al., 2014) and neural stem cell self-renewal (Horvath et al., 2006; Paik et al., 2009). To monitor the long-term fate of ASPM-expressing cells *in vivo* during neurogenesis, Marinaro et al. generated *Aspm-CreER^T2^*/*Nestin-GFP^flox^*-TK mice using the Cre-LoxP system. Tamoxifen was injected into mice at E12.7 and E13.2 to activate the thymidine kinase (TK) gene, and ganciclovir was administered from E14.5 until E18.5 to selectively kill ASPM-positive/TK-positive cells. The mice exhibited severe impairments in forebrain development, SVZ cell proliferation, and the laminar organization of the cortex (Marinaro et al., 2011). ASPM is also required for the orientation of dividing progenitors and neuronal migration in mouse neocortex. Knockdown of *Aspm* in the telencephalic hemisphere of E10.5 or E12.5 mice *via* endoribonuclease-prepared, short interfering RNAs altered the orientation of the neuroepithelial cleavage plane, causing it to become less perpendicular to the ventricular surface (Fish et al., 2006). In addition, using *Aspm^−/−^* mice generated by Cre-loxP-mediated deletion of exons 2 and 3, Fujimori et al. found disruption of cortical layer-specific transcription factor expression (Satb2, Ctip2, Tbr1) in E16.5 embryos and a thinner cortical layer VI in the adult neocortex, suggesting that loss of ASPM impaired neuronal differentiation (Fujimori et al., 2014). The effects of ASPM depletion, namely altered differentiation, premature cell cycle exit, and apoptosis ultimately reduce cerebellar growth (Williams et al., 2015).

In addition to its role in embryogenesis, ASPM is also involved in neurogenesis in adult mouse tissues. For example, in P30 mouse SVZ, descendants of Aspm-positive cells were shown to promote the generation of neurons, astrocytes, and cells of oligodendrocyte lineage (Marinaro et al., 2011). Moreover, cerebral organoid culture *in vitro* to generate human brain-like organs has advanced research into human brain disease, particularly with regard to neurogenesis in the developing neocortex (Li et al., 2017). For example, using patient-specific induced pluripotent stem cells with a dysfunctional *ASPM* gene to generate cerebral organoids, Li et al. found loss of luminal structures and neural precursors, consistent with the *Aspm^−/−^* mouse phenotype (Li et al., 2017).

Bond et al. firstly reported human ASPM as a determinant of cerebral cortical size, suggesting that brain size is partially modulated by its mitotic spindle activity (Bond et al., 2002). The correlation between ASPM and brain size has been confirmed in multiple species, including humans, mice, zebrafish, and ferret (Bond et al., 2003; Kim et al., 2011; Johnson et al., 2018; Ogi et al., 2018). Individuals with *ASPM* mutations may have variable levels of delayed development in various areas, such as motor, speech, and language skills, and cognitive abilities (Naseer et al., 2020; Liaci et al., 2021). Therefore, understanding the functions of ASPM in neurogenesis is crucial. Notably, the clinical manifestations of ASPM-related microcephaly can vary among individuals and may be influenced by the specific mutation present. Numerous mutations in the *ASPM* gene have been identified in MCPH patients (Table 1), some of which have been incorporated into different animal models seeking to investigate the pathological mechanisms of ASPM mutation in microcephaly. Below, we summarize the detailed functions of ASPM in several animal models.

**Table 1:** Mutations in human ASPM in patients diagnosed with MCPH.

Location (exon)	cDNA mutation	Protein mutation	Mutation type	Homozygosity	References
18	c.6012_6013delTA	p. Tyr2004*	Nonsense		Xu et al. (2022)
18	c.6015_6016delGG	p. Arg2005Serfs*48	Frameshift		
16	c.3978G > A	p.Trp1326*	Nonsense		Hussain et al. (2022)
17	c.4019delA	p.Lys1340Argfs*29	Frameshift	Heterozygous	Li et al. (2022)
3	c.1789C > T	p.Arg597*	Nonsense	Heterozygous	
6	c.2525_2531delGTGATGT	p.Ser842fs*9	Frameshift	Heterozygous	
18	c.6994C > T	p.Arg2332*	Nonsense	Heterozygous	
18	c.7782_7783delGA	p.Lys2595Serfs*6	Frameshift	Heterozygous	Nicholas et al. (2009), Passemard et al. (2009), Tan et al. (2014)
18	c. 8214dupT	p.Q2739fs	Frameshift	Heterozygous	Zhang et al. (2022)
23	c. 9541C > T	p.R3181X	Nonsense	Heterozygous	
18	c.5477_5478del	p.Ile1826Serfs*4	Frameshift	Homozygous	von Wrede et al. (2022)
18	c.5219_5225delGAGGATA	p.Arg1740Thrfs*7	Frameshift	Homozygous	McSherry et al. (2018)
18	c.7792C > T	p. Gln2598*	Nonsense	Homozygous	Turkyilmaz and Sager (2022)
18	c.6854_6855del	p.(Leu2285GlnfsTer32)	Frameshift		Naqvi et al. (2022)
25	c.10097_10098delGA	p.(Gly3366Glufs*19)	Frameshift	Homozygous	Makhdoom et al. (2022)
18	c.4174C > T	p.(Arg1392Ter)	Nonsense	Homozygous	Correia-Costa et al. (2022)
18	c.8862dupA	p.V2955Sfs*12	Frameshift	Homozygous	Bolat et al. (2022)
17	c.4162dupA	p.1388 fs*4	Frameshift	Homozygous	
2	c.646G > T	p.E216*	Nonsense	Homozygous	
3	c.1615_1616del	p. Glu539ArgfsTer15	Frameshift	Heterozygous	Tran et al. (2021)
1	c.∗293 T > A	p. Leu98Ter	Frameshift	Heterozygous	
17	c.3877_3880delGAGA	p.Glu1293Lysfs*10	Frameshift	Homozygous	Batool et al. (2023)
22	c. 9601C > T	p.(Gln3201*)	Nonsense	Homozygous	Makhdoom et al. (2021)
3	c.719_720delCT	p.(Ser240Cysfs*16)	Frameshift	Homozygous	
21	c.9492 T > G	p.(Tyr3164*)	Nonsense	Homozygous	Muhammad et al. (2009), Kousar et al. (2010), Sajid Hussain et al. (2013)
3	c.727C > T	p.(Arg243*)	Nonsense	Homozygous	Rasool et al. (2020)
3	c.1602_1605delTCAA	p.(Asn534Lysfs*14)	Frameshift	Homozygous	
3	c.1615_1616delGA	p.(Glu539Argfs*15)	Frameshift	Homozygous	
13	c.3193C > T	p.(Gln1065*)	Nonsense	Homozygous	
18	c.8718_8721delTTTA	p.(Leu2907Argfs*30)	Frameshift	Homozygous	
23	c.9601C > T	p.(Gln3201*)	Nonsense	Homozygous	
25	c.9961C > T	p.(Gln3321*)	Nonsense	Homozygous	
18	c.6854_6855delTC	p.(Leu2285Glnfs*32)	Frameshift	Heterozygous	
25	c.9976_9977dupGT	p.(Ser3327 Tyrfs*14)	Frameshift	Heterozygous	
15	c.3741G > A	p.(Lys1247=)	Substitution	Heterozygous	
9	c.2738dupT	p.Cys914fs	Frameshift	Homozygous	Bazgir et al. (2019)
16	c.3978G > A	p.Trp1326*	Missense	Homozygous	Ahmed et al. (2019)
18	c.7782_7783delGA	p.(Lys2595Serfs*6)	Frameshift	Heterozygous	
9	c.2936 + 5G > A	(IVS9 + 5G > A)	Frameshift	Homozygous	
23	c.9742_9745del	p.Lys3248Serfs*13	Frameshift	Heterozygous	Okamoto et al. (2018)
18	c.7543C > T	p.Arg2515Ter	Substitution	Homozygous	Khan et al. (2018)
3	c.1850_1853de	p.Thr617Lysfs*30	Frameshift	Homozygous	Létard et al. (2018)
4	c.1932del	p.Phe645Serfs*23	Frameshift	Homozygous	
4	c.1943_1944insC	p.Ile649Asnfs*3	Frameshift	Homozygous	
9	c.2638G > T	p.Glu880*	Nonsense	Homozygous	
intron10	c.2936 + 2 T > C	p.?	Splicing	Homozygous	Létard et al. (2018)
13	c.3185_3189del	p.Asn1062Argfs*28	Frameshift	Homozygous	
13	c.3269dup	p.Asp1091*	Nonsense	Homozygous	
intron15	c.3741 + 3A > G	p.?	Splicing	Homozygous	
18	c.4250_4251del	p.Tyr1417*	Nonsense	Homozygous	
18	c.4732C > T	p.Arg1578*	Nonsense	Homozygous	
18	c.4806 T > G	p.Tyr1602*	Nonsense	Homozygous	
18	c.4992_4996dup	p.Arg1667Ilefs*12	Frameshift	Homozygous	
18	c.5590_5591del	p.Leu1864Serfs*2	Frameshift	Homozygous	
18	c.5886_5887del	p.Leu1963Glufs*9	Frameshift	Homozygous	
18	c.5940del	p.Tyr1981Ilefs*13	Frameshift	Homozygous	
18	c.6513dup	p.Val2172Serfs*7	Frameshift	Heterozygous	
18	c.6568C > T	p.Gln2190*	Nonsense	Heterozygous	
18	c.6658C > T	p.Gln2220*	Nonsense	Homozygous	
18	c.6919C > T	p.Gln2307*	Nonsense	Homozygous	
18	c.6920_6921del	p.Gln2307Leufs*10	Frameshift	Homozygous	
18	c.7744del	p.Ile2582Serfs*34	Frameshift	Homozygous	
18	c.7753G > T	p.Glu2585*	Nonsense	Homozygous	
18	c.8599delinsAT	p.Gln2867Ilefs*5	Frameshift	Homozygous	
18	c.8700_8702delinsCC	p.Lys2900Asnfs*38	Frameshift	Homozygous	
18	c.8702del	p.His2901Leufs*37	Frameshift	Heterozygous	
20	c.9069_9075del	p.His3023Glnfs*2	Frameshift	Homozygous	
23	c.9446_9447del	p.Arg3149Metfs*17	Frameshift	Homozygous	
28	c.10369del	p.Glu3457Lysfs*13	Frameshift	Homozygous	
3	c.1386delC	p.Tyr462*	Nonsense	Homozygous	Marakhonov et al. (2018)
13	c.3384_3385	p.Lys1129Ter	Frameshift	Homozygous	Bhargav et al. (2017)
3	c.1235_1239delAAGTA	p.Lys412Thrfs*5	Frameshift	Homozygous	Ahmad et al. (2017)
6	c.2420delG	p.Gly807Glufs*7	Frameshift	Homozygous	
13	c.3491_3494delGTAC	p.Arg3491Leufs*15	Frameshift	Homozygous	
17	c.4212G > A	p. Trp1404*	Nonsense	Homozygous	
18	c.8098C > T	p.Arg2700*	Nonsense	Homozygous	
18	c.6851_6854delTCTC	p.Leu2285Argfs*6	Frameshift	Heterozygous	
18	c.7129C > T	p.Gln2377*	Nonsense	Heterozygous	
18	c.5959C > T	p.Gln1987*	Nonsense	Heterozygous	
18	c.8508_8509delGA	p.Lys2837Metfs*34	Frameshift	Homozygous	Bond et al. (2003), Gul et al. (2006), Muhammad et al. (2009)
24	c.10013delA	p.Asp3338Valfs*2	Nonsense	Homozygous	Khan et al. (2017)
23	c.9730C > T	p.Arg3244*	Frameshift	Homozygous	
17	c.3978G > A	p.W1326*	Nonsense	Homozygous/ Heterozygous	Wang et al. (2017)
18	c.4185G > A	p.W1395*	Nonsense	Heterozygous	
18	c.6994C > G	p. R2332*	Nonsense	Homozygous	
23	c.9557C > G	p.S3186*	Nonsense	Homozygous	Bond et al. (2002, 2003), Sajid Hussain et al. (2013)
16	c.3742-1G > C	Lys1247Glyfs*9	Splice-site	Homozygous	Hashmi et al. (2016)
18	c.5149delA	p. Ile1717fsx1	Frameshift	Heterozygous	Choi et al. (2016)
3	c.688delG	p.E230Nfs*3	Frameshift		Abdel-Hamid et al. (2016)
3	c.1789C > T	p.R597*	Nonsense		
22	c.9541C > T	p.R3181*	Nonsense		
9	c.2936 + 1G > A	VS10þ1G > A	Splice-site		
10	c.3108_3114delTGTGGAT	p.V1037Gfs*13	Frameshift		
16	c.3979C > T	p.R1327*	Nonsense		
18	c.4612C > T	p.R1538*	Nonsense		
22	c.9541C > T	p.R3181*	Nonsense		
3	c.1959_1961delCAAA	p.N653Kfs*14	Frameshift		Bond et al. (2003), Tan et al. (2014), Abdel-Hamid et al. (2016)
19	c.9190C > T	p.R3064*	Nonsense	Homozygous	Nicholas et al. (2009), Bond et al. (2002), Abdel-Hamid et al. (2016)
24	c.9697C > T	p.R3233*	Nonsense	Homozygous	Tan et al. (2014), Muhammad et al. (2009), Abdel-Hamid et al. (2016)
9	c.2967G > A	p.W989*	Nonsense	Homozygous	Kraemer et al. (2016)
18	c.8200_8201delAA	p.N2734Lfs*16	Frameshift	Homozygous	
22	c.9539A > C	p.Q3180P	Missense	Homozygous	
9	c.2938C > T	p.R980*	Nonsense	Homozygous	
18	c.5606_5607insC	p.H1870Tfs*26	Frameshift	Homozygous	
18	c.6750delT	p.F2250Lfs*10	Frameshift	Heterozygous	Nakamura et al. (2015)
13	c.3327 T > G	p.Tyr1109*	Nonsense	Heterozygous	Tan et al. (2014)
24	c.9910C > T	p.Arg3304*	Nonsense	Heterozygous	
3	c.637del	p.Ile213Tyrfs*47	Frameshift	Homozygous	
18	c.8017C > T	p.Gln2673*	Nonsense	Homozygous	
17	c.3853_3854del	p.Asp1285Serfs*32	Frameshift	Heterozygous	
18	c.7308dup	p.Val2437Cysfs*14	Frameshift	Heterozygous	
18	c.5196 T > A	p.Cys1732*	Nonsense	Heterozygous	
23	c.9454C > T	p.Arg3152*	Nonsense	Heterozygous	
18	c.7612C > T	p.Gln2538*	Nonsense	Homozygous	
10	c.2791C > T	p.Arg931*	Nonsense	Homozygous	
3	c.803_804del	p.Lys268Serfs*4	Frameshift	Heterozygous	
13	c.3390 + 3_6del		Splicing	Heterozygous	Tan et al. (2014)
1	c.117_118del	p.Leu41Glnfs*30	Frameshift	Homozygous	
18	c.8133_8136del	p.Lys2712Leufs*16	Frameshift	Heterozygous	
22	c.9309_9310del	p.Arg3103Serfs*20	Frameshift	Heterozygous	
18	c.7665del	p.Ala2556Leufs*4	Frameshift	Heterozygous	
18	c.7825C > T	p.Gln2609*	Nonsense	Heterozygous	
17	c.3960_3961insA	p.Val1321Serfs*29	Frameshift	Heterozygous	
3	c.1726_1729del	p.Lys576Alafs*10	Frameshift	Heterozygous	
10	c.2936dup	p.Arg980Alafs*31	Frameshift	Heterozygous	
6	c.2419 + 2 T > C	p.Leu3035*	Splicing	Heterozygous	
21	c.9104 T > A		Nonsense	Heterozygous	
3	c.1138C > T	p.Gln380*	Nonsense	Homozygous	
18	c.8711_8712del	p.Gln2904Argfs*15	Frameshift	Homozygous	
11	c.2968del	p.Asp990Thrfs*11	Frameshift	Heterozygous	
18	c.4728_4729del	p.Arg1576Serfs*7	Frameshift	Heterozygous	
19	c.8903G > A	p.Trp2968*	Nonsense	Homozygous	
18	c.7857dup	p.Glyn2620Thrfs*17	Frameshift	Homozygous	
8	c.2571G > A	p.Trp857*	Nonsense	Heterozygous	
18	c.8227C > T	p.R2743X	Nonsense	Heterozygous	Hu et al. (2014)
18	c.7772_7775delAAAA	p.2591 fs	Frameshift	Heterozygous	
18	c.4849C > T	R1617X	Nonsense	Homozygous	Papari et al. (2013)
17	c.3979C > T	p.Arg1327*	Nonsense	Homozygous	Sajid Hussain et al. (2013)
18	c.6131C > T	p.Gln2051*	Nonsense	Homozygous	
14	c.3796G > T	p.E1266X	Nonsense	Heterozygous	Nicholas et al. (2009), Ariani et al. (2013)
18	c.7815_7816del	p.E2605fs	Frameshift	Heterozygous	
18	c.5188G > T	p.Glu1730X	Nonsense	Homozygous	Darvish et al. (2010)
18	c.5584A > C	p.Lys1862Gln	Missense		
21	c.9286C > T	p.Arg3096X	Nonsense	Homozygous	
13	c.3229_3230delAA	p.Lys1077fs	Frameshift		
Intron15	c.3741 + 1G > A	Truncated protein	Splicing		
14	c.3505_3506delGT	p.Val1169fs	Frameshift		
21	c.9091C > T	p.Arg3031X	Nonsense	Homozygous	
Intron1	c.297 + 1G > C	Truncated protein	Splicing		
14	c.3506_3507delTG	p.Val1169fs	Frameshift		
11	c.3055C > T	p.Arg1019X	Nonsense	Homozygous	Muhammad et al. (2009), Nicholas et al. (2009), Darvish et al. (2010)
22	c.9319C > T	p.Arg3107X	Nonsense	Homozygous	Muhammad et al. (2009), Passemard et al. (2009), Darvish et al. (2010)
24	c.10060C > T	p.Arg3354X	Nonsense		Halsall et al. (2010)
17	c.3977G > A	p.Trp1326X	Nonsense		
17	c.4184G/A	p.Trp1395X	Nonsense		
18	c.7569_7570delAA	p.Gln2523fs	Nonsense		
3	c.2101C > T	p.Q701X	Nonsense	Homozygous	Kousar et al. (2010)
18	c.6686delGAAA	p.R2229TfsX9	Frameshift	Homozygous	Passemard et al. (2009), Kousar et al. (2010)
1	c.77delG	p.G26AfsX41	Frameshift	Homozygous	
5	c.2389C > T	p.Arg797X	Nonsense	Heterozygous	Passemard et al. (2009), Saadi et al. (2009)
18	c.7781_7782delAG	p.Gln2594fsX6	Frameshift	Heterozygous	Passemard et al. (2009)
13	c.3477_3481delCGCTA	p.A1160fs	Frameshift	Homozygous	Muhammad et al. (2009)
18	c.6732delA	p.Y2245fs	Frameshift	Homozygous	
23	c.9677_9678insG	p.C3226fs	Frameshift	Homozygous	
22	c.9595A > T	p.K3199X	Nonsense	Homozygous	
18	c.8668C > T	p.Q2890X	Nonsense	Homozygous	
1	c.74delG	p.Arg25fs	Nonsense		Nicholas et al. (2009)
1	c.297 + 1460_3391-242del21844	Truncated protein	Splicing		
1	c.440delA	p.Lys147fs	Frameshift		
2	c.577C > T	p.Gln193X	Nonsense		
3	c.1152_1153delAG	p.Ser384fs	Frameshift	Homozygous	
3	c.1179delT	p.Pro393fs	Frameshift		
3	c.1366G > T	p.Glu456X	Nonsense		
3	c.1406_1413delATCCTAAA	p.Asn469fs	Frameshift		
3	c.1590delA	p.Lys530fs	Frameshift		
8	c.2761-25A > G	Truncated protein	Splicing		
11	c.3188 T > G	p.Leu1063X	Nonsense	Homozygous	
14	c.3710C > G	p.Ser1237X	Nonsense	Homozygous	
18	c.4855_4856delTA	p.Tyr1619fs	Frameshift	Homozygous	
18	c.7489_7493delTATAT	p.Tyr2497fs	Frameshift		
18	c.7782_7783delGA	p.Gln2594fs	Frameshift	Homozygous	
18	c.7859_7860delAG	p.Gln2620fs	Frameshift		
18	c.8130_8131delAA	p.Thr2710fs	Frameshift		
18	c.8378delT	p.Met2793fs	Frameshift	Homozygous	
18	c.8844delC	p.Ala2948fs	Frameshift		
3	c.1959_1961delCAAA	p.Asn653fs	Frameshift		Bond et al. (2003), Tan et al. (2014)
18	c.6335_6336delAT	p.His2112fs	Frameshift	Homozygous	Trimborn et al. (2004), Nicholas et al. (2009)
18	c.7761 T > G	p.Tyr2587X	Nonsense	Homozygous	Nicholas et al. (2009), Bond et al. (2002)
19	c.9178C > T	p.Gln3060X	Nonsense	Homozygous	Kumar et al. (2004), Nicholas et al. (2009),Tan et al. (2014)
20	c.9238A > T	p.Leu3080X	Nonsense	Homozygous	Nicholas et al. (2009)
23	c.9681delA	p.Thr3227fs	Frameshift	Homozygous	
23	c.9745_9746delCT	p.Leu3249fs	Frameshift	Homozygous	
23	c.9789 T > A	p.Tyr3263X	Nonsense	Homozygous	
1	c.349C > T	p.Arg117X	Nonsense	Homozygous	Bond et al. (2002), Kumar et al. (2004)
3	c.719_720delCT	p.Ser240fs	Frameshift	Homozygous	Bond et al. (2002, 2003)
3	c.1727_1728delAG	p.Lys576fs	Frameshift		Bond et al. (2003)
4	c.1990C > T	p.Gln664X	Nonsense	Homozygous	Bond et al. (2003)
Intron7	c.2936 + 5G > T	Removes splice donor site, additional 2 aa then stop	Splicing	Homozygous	Bond et al. (2003)
11	c.3082G > A	Removes splice donor site, additional 3 aa then stop	Splicing	Homozygous	Bond et al. (2003)
14	c.3527C > G	p.Ser1176X	Nonsense		Bond et al. (2003)
15	c.3663delG	p.Arg1221fs	Frameshift	Homozygous	Bond et al. (2003)
18	c.4581delA	p.Gly1527fs	Frameshift	Homozygous	Bond et al. (2003)
18	c.4795C > T	p.Arg1599X	Nonsense	Homozygous	Bond et al. (2003), Tan et al. (2014)
18	c.5136C > A	p.Tyr1712X	Nonsense	Homozygous	Bond et al. (2003), Gul et al. (2007)
21	c.9159delA	p.Lys3053fs	Frameshift	Homozygous	Bond et al. (2002), Bond et al. (2003), Kousar et al. (2010)
24	c.9754delA	p.Arg3252fs	Frameshift		Bond et al. (2003)
Intron25	c.9984 + 1G > T	Removes splice donor site, additional 29 novel aa then stop	Splicing	Homozygous	Bond et al. (2003)
16	c.3811C > T	p.Arg1271X	Nonsense	Homozygous	Bond et al. (2003), Nicholas et al. (2009), Passemard et al. (2009)
18	c.6189 T > G	p.Tyr2063X	Nonsense	Homozygous	Shen et al. (2005), Tan et al. (2014)
19	c.9118_9119insCATT	p.Tyr3040fs	Frameshift	Homozygous	Gul et al. (2006)
3	c.1260_1266delTCAAGTC	p.Ser420fs	Frameshift	Homozygous	Gul et al. (2006), Kousar et al. (2010)
26	c.10059C > A	p.Tyr3353X	Nonsense	Homozygous	Gul et al. (2007)

### 3.2. Animal models for ASPM

To better understand the function of ASPM in the development of the cerebral cortex and other organs, several studies have used different genetic approaches in various species (*Drosophila*, mice, ferrets, and zebrafish) to edit *ASPM*, based on human mutations. These studies have uncovered the molecular mechanisms underlying the microcephaly phenotype (Gonzalez et al., 1988, 1990; Bond et al., 2003; Pulvers et al., 2010; Kim et al., 2011; Fujimori et al., 2014; Johnson et al., 2018; Ogi et al., 2018; Mori et al., 2022).

#### 3.2.1. Drosophila

*Drosophila*, which has a similar neurodevelopment with human, acts as a desirable animal model to study human neurodevelopmental disorders such as microcephaly (Robinson et al., 2020). From centrosome studies in *Drosophila*, many human microcephaly genes were originally identified including *Asp*, *merry-go-round (mgr)* and *polo* (Ripoll et al., 1985; Gonzalez et al., 1988; Sunkel and Glover, 1988; Singh et al., 2014; Jana et al., 2016; Ramdas Nair et al., 2016). *Drosophila* syncytial embryos and *larvae with Asp* mutants exhibited high mitotic index (MI) and notable presence of hyperploid and polyploid cells (Ripoll et al., 1985; Gonzalez et al., 1990). Lately, *Asp* was been found as a microtubule-binding protein that localizes to the mitotic spindle polar and maintain the spindle stability in *Drosophila* (Saunders et al., 1997; Gonzalez et al., 1998; do Carmo Avides and Glover, 1999). Downregulation of *Asp* by siRNA in S2 cells caused increased mitotic index, loss of spindle pole focus and detached centrosomes (Morales-Mulia and Scholey, 2005). Furthermore, Schoborg et al. and Goshima et al. found the activity of *Asp* is regulated by calmodulin (CaM), interaction of both is required for focused spindle pole and centrosome detachment (Goshima et al., 2007; Schoborg et al., 2015). To analyze the *Asp* function in neural development, Rujano et al. characterized a *Asp* mutant (2,396–2,402 bp missing) with a premature stop codon at amino acid 721 and found defects in the brain size and neuroepithelium morphogenesis (Rujano et al., 2013).

#### 3.2.2. Mice

In mouse embryonic stem (ES) cells, gene trapping is an efficient method of genome mutagenesis that can help to elucidate the roles of genes in specific biological pathways (Friedel and Soriano, 2010). Utilizing this technique, scientists generated mice with various ASPM truncations from gene trap ES cells: AJ0069 (AspmGt(AJ0069)Wtsi), in which ASPM was truncated between exons 25 and 26 of ASPM; AA0137 (AspmGt(AA0137)Wtsi), in which ASPM was truncated between exons 7 and 8; and ASPM SA/SA mice, in which ASPM was truncated between exons 6 and 7 (Pulvers et al., 2010; Williams et al., 2015). Among the mutated mice, both AspmGt(AJ0069)Wtsi and AspmGt(AA0137)Wtsi-hom (homozygotes) showed reduced brain weight in P0.5 day neonates and 8- to 12-week-old adults, while ASPM SA/SA mice showed reduced brain and cerebellar weight at P30W. The microcephaly phenotype in AspmGt(AA0137)Wtsi-hom mice was rescued by expression of human ASPM, indicating the specific role of ASPM mutation in microcephaly.

Two studies using similar strategies to generate CAG-driven Cre-loxP conditional *Aspm* knockout mice showed decreased fractional anisotropy (FA) values which is mostly used to quantify white matter integrity in the cortex and the changes of FA were closely correlated with neuropathology, including abnormal neurite outgrowth and differentiation, white matter at P5W, reduced brain size in the neocortex, thinner cortical layer VI, and significantly reduced testis weight at P12W, compared with *Aspm^+/+^* mice (Fujimori et al., 2014; Ogi et al., 2018). In addition, in mouse embryos, Martínez et al. recently used CRISPR-Cas9 technology to insert a stop codon into exon 3 of *Aspm*, which partially reduced ASPM levels at the centrosome and caused mild microcephaly, with decreased brain weight and volume at P30 (González-Martínez et al., 2021).

Notably, in addition to microcephaly, mice carrying *Aspm* mutations showed decreased fertility, with reductions in testicular size, oocyte number, ovarian weight, pregnancy rate, and offspring number. In addition, in female conditional *Aspm* knockouts, ovary size was reduced, and there were lower numbers of developing follicles during postnatal maturation and aging, suggesting the crucial role of ASPM in ovarian development (Pulvers et al., 2010; Mori et al., 2022).

It is worth noting, however, that some studies have shown a milder form of microcephaly in mutated mice, compared with that observed in human microcephaly patients. This reduced severity may be due to differences in brain size, gyrification, and progenitor divisions in mice and humans (Pulvers et al., 2010; Johnson et al., 2018).

#### 3.2.3. Ferret

Due to the limited effect of *Aspm* mutations in mice and the considerable differences between mouse and human brains, some studies have used the ferret as an alternative model animal. Ferrets have a larger, gyrified cortex and greater NPC diversity than mice, and ferret ASPM shares a greater level of homology with the human ASPM (Fietz et al., 2010). Johnson et al. generated *Aspm* knockout ferrets by carrying out genome editing to target exon 15 with a mutation identified in a previous study (Bond et al., 2003; Johnson et al., 2018). *Aspm* knockout ferrets showed robust microcephaly, with a reduction in brain weight of around 25–40%. This reduction reflected the loss of cortical units caused by the premature translocation of ventricular radial glial cells to the outer SVZ (Johnson et al., 2018). These findings suggested that ASPM controls the progress of cortical expansion, thus ensuring normal brain development.

#### 3.2.4. Zebrafish

In zebrafish, Kim et al. knocked down *aspm* by using morpholino antisense oligonucleotides (MO) to target the exon 11 splice donor site, thereby blocking translation (Kim et al., 2011). In the mutated zebrafish, brain size was reduced at 35 h post-fertilization, and the cells showed mitotic arrest followed by apoptotic cell death (Kim et al., 2011). These findings underscore the importance of ASPM in regulating brain development across different species and highlight the correlation between mitotic function and early brain development.

Overall, these findings support the notion that ASPM plays a significant role in neurogenesis by maintaining the pool of NPCs and regulating their differentiation. It has been suggested that positive selection of ASPM may have contributed to the evolutionary expansion of the human brain (Gonzalez et al., 1988, 1990; Mori et al., 2022).

## 4. ASPM regulates genome stability

While the growth inhibition in cerebellar and medulloblastoma has been confirmed in ASPM knockout animal models, an increase of DNA damage and apoptosis was also noted (Williams et al., 2015). This suggested that ASPM may have functions in maintaining genome stability and cell survival.

### 4.1. ASPM in DNA replication

DNA replication is an essential cellular event that favors cell growth and proliferation. Increasing numbers of studies have shown that defects in DNA replication initiation and replication stress cause cortical malformations such as microcephaly (Bicknell et al., 2011; de Munnik et al., 2012; Jackson et al., 2014; Reynolds et al., 2017; Jayaraman et al., 2018). Some microcephaly genes are also involved in DNA replication. For example, the microcephaly gene *DONSON* encodes a replication fork protein that maintains genome stability by stabilizing stalled forks and activating replication checkpoints (Reynolds et al., 2017). Several bioinformatic methods have indicated that ASPM, along with its upstream regulator trophinin-associated protein (TROAP) and downstream factor cell division cycle 20 (CDC20), may regulate cell replication during the S and G2 cell cycle phases; however, details of the mechanism involved remain unknown (Liu et al., 2022). Recently, a study carried out by our group in HeLa cells uncovered the function of ASPM in maintaining genome stability in response to replication stress (Wu et al., 2022). We identified potential ASPM interaction partners from mass spectrum data, including several DNA replication factors, such as mini-chromosome maintenance protein 5 (MCM5), replication factor complex (RFC1-5), and replication protein A (RPA1, RPA2). Nonetheless, in *ASPM* knockout cells, we did not observe any differences in replication speed or percentages of cells in S phase, suggesting that ASPM is dispensable for normal replication. By contrast, following replication stress induced by hydroxyurea or aphidicolin, ASPM stabilized replication forks and antagonized the degradation of nascent DNA strands mediated by meiotic recombination 11 (MRE11) nuclease. Loss of ASPM also resulted in reduced activation of the ATR-CHK1 signaling pathway (Wu et al., 2022). This work provides new insights to enhance our understanding of the pathogenesis of ASPM loss in diseases such as microcephaly and cancer.

### 4.2. ASPM in DNA damage response

Diseases such as neurodevelopmental disorders, neurodegenerative disorders, aging-related conditions, and cancer are commonly caused by gene alterations arising from DNA damage, replication errors, chromosomal segregation defects, and other factors. Genomic DNA is constantly threatened by endogenous or exogenous factors, and efficient DNA damage repair is crucial for maintaining genome stability. Impairment of DNA damage repair leads to genetic alterations and can also cause microcephaly if it occurs during neural development (O'Driscoll and Jeggo, 2008; Zhou et al., 2013; Shiwaku and Okazawa, 2015; Wei et al., 2016; Martins et al., 2022). In *Drosophila*, DNA double-strand breaks caused by ionizing radiation (IR) treatment reportedly induced microcephaly by promoting the premature differentiation of neural stem cells and neuroblasts, without affecting apoptotic cell death (Barazzuol et al., 2017; Wagle and Song, 2020). Loss of ASPM also increased DNA damage in cerebellar granule neuron progenitors in mice (Williams et al., 2015), while ASPM expression was downregulated in IR-treated human cells (embryonic lung fibroblasts, HeLa, and MCF7), mouse embryonic brain cells, and neurospheres (Fujimori et al., 2008). The latter finding could explain the mechanism of microcephaly formation caused by IR (Fujimori et al., 2008). Moreover, ASPM loss sensitized glioblastoma cells (U87MG), cervical cancer cells (HeLa), and normal human fibroblasts (AG1521) to X-irradiation, H_2_O_2_, camptothecin, and increased chromosomal aberrations arising from impaired DNA repair (Kato et al., 2011). This sensitization may result from reduced levels of breast cancer type 1 susceptibility protein (BRCA1), a key factor involved in homologous recombination repair, but the precise mechanism is yet to be determined (Zhong et al., 2005). Recently, we demonstrated that ASPM is recruited to DNA damage sites, where it protects BRCA1 from degradation by antagonizing ubiquitination mediated by HECT domain and RCC1-Like domain-containing protein 2 (HERC2, an E3 ligase; Xu et al., 2021). By promoting efficient homologous recombination, ASPM maintained chromosome stability following X-ray-induced damage. However, further research is needed to enhance our understanding of the role of ASPM-related DNA repair in neurological disorders such as microcephaly.

### 4.3. ASPM in cancer

Cancer is a severe disease characterized by high rates of cell proliferation and continuous cell division. As an essential gene involved in regulating cell division, ASPM also contributes to cancer development. Wnt/β-catenin signaling, which is important for cell proliferation, organogenesis, tissue homeostasis, and embryonic development, is frequently activated in cancer cells (Schunk et al., 2021; Yu et al., 2021). Recently, studies demonstrated that ASPM interacts with and stabilizes disheveled-3 (Dvl-3), a cardinal upstream regulator of the Wnt signaling pathway. This interaction increases Wnt-induced β-catenin transcriptional activity, promoting proliferation, stemness properties, and tumorigenicity in prostate cancer cells, anaplastic thyroid cancer cells, and glioblastoma cells (Pai et al., 2019; Chen et al., 2020; Jiang et al., 2022). In addition, in a rapidly tumorigenic medulloblastoma mouse model, *Aspm* knockout significantly slowed medulloblastoma growth and increased DNA damage, suggesting that ASPM promotes tumorigenesis (Williams et al., 2015).

**Figure 3:**
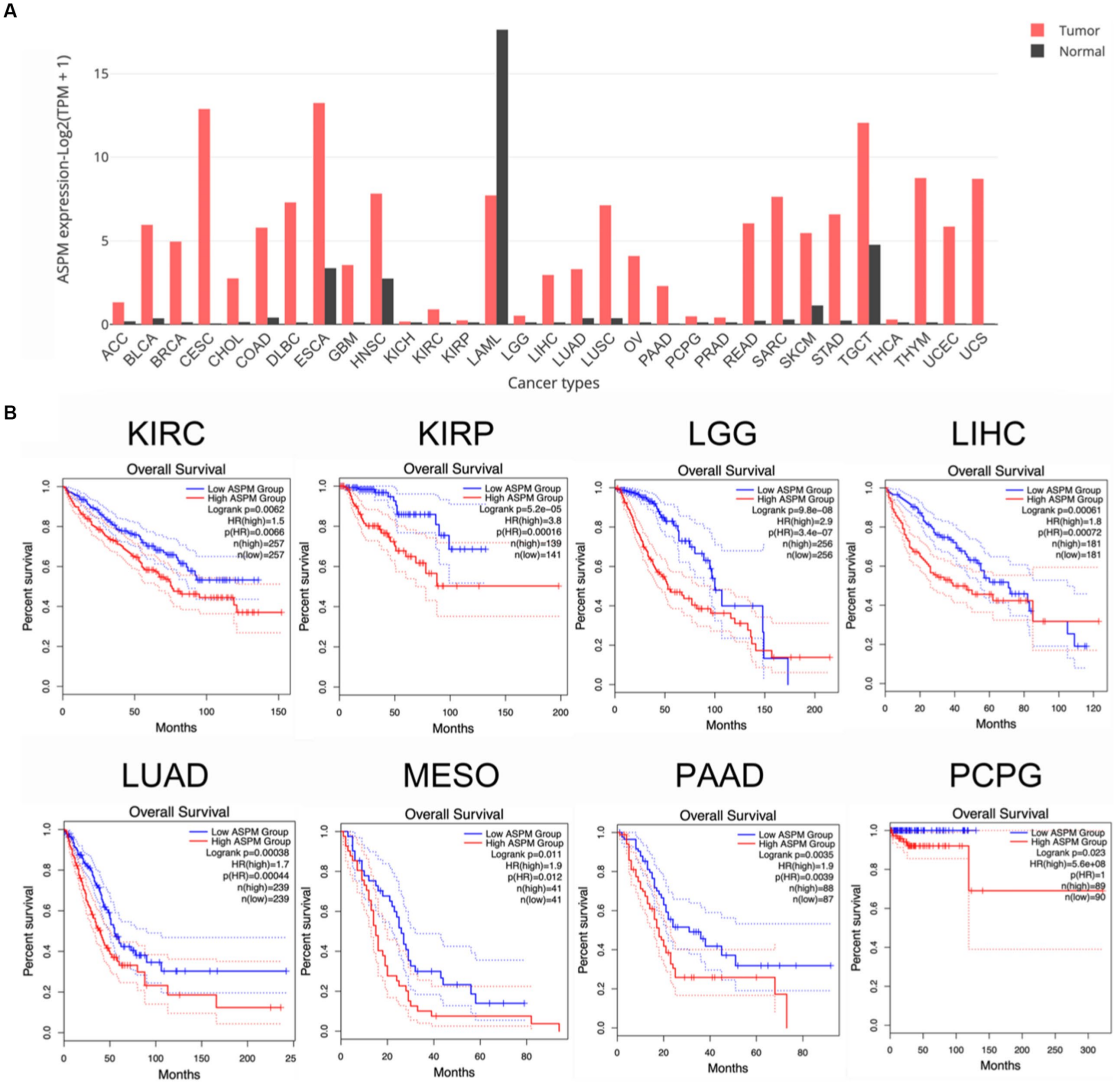
High levels of *ASPM* expression are associated with poor prognosis in various cancers based on The Cancer Genome Atlas and Gene Expression Profiling Interactive Analysis databases. **(A)** Bar plot of *ASPM* gene expression profiles for tumor samples and normal tissues. ACC, adrenocortical carcinoma; BLCA, bladder urothelial Carcinoma; BRCA, breast invasive carcinoma; CESC, cervical squamous cell carcinoma and endocervical adenocarcinoma; CHOL, cholangio carcinoma; COAD, colon adenocarcinoma; DLBC, diffuse large B-cell lymphoma; ESCA, esophageal carcinoma; GBM, glioblastoma multiforme; HNSC, head and neck squamous cell carcinoma; KICH, kidney chromophobe; KIRC, kidney renal clear cell carcinoma; KIRP, kidney renal papillary cell carcinoma; LAML, acute myeloid leukemia; LGG, brain lower grade glioma; LIHC, liver hepatocellular carcinoma; LUAD, lung adenocarcinoma; LUSC, lung squamous cell carcinoma; OV, ovarian serous cystadenocarcinoma; PAAD, pancreatic adenocarcinoma; PCPG, pheochromocytoma and paraganglioma; PRAD, prostate adenocarcinoma; READ, rectum adenocarcinoma; SARC, sarcoma; SKCM, skin cutaneous melanoma; STAD, stomach adenocarcinoma; TGCT, testicular germ cell tumor; THCA, thyroid carcinoma; THYM, thymoma; UCEC, uterine corpus endometrial carcinoma; UCS, uterine carcinosarcoma. **(B)** High levels of ASPM expression in different types of cancers are correlated with poor prognosis. Survival analysis was performed online (http://gepia.cancer-pku.cn) to analyze correlations between ASPM levels and overall survival in cancers.

Based on The Cancer Genome Atlas database,^1^ comparison of tumor and normal tissues shows the upregulation of ASPM expression in many tumors (Figure 3A). Genomic analysis has also identified multiple *ASPM* mutations (Table 2) in tumors.^2^ Meanwhile, high levels of *ASPM* expression correlate with poor prognosis in various types of cancer (Figure 3B), including bladder cancer (Chen et al., 2019, 2021; Gao et al., 2020; Liu et al., 2023), prostate cancer (Xie et al., 2017; Pai et al., 2019; Xu et al., 2019), breast cancer (Shubbar et al., 2013; Tang et al., 2019; Wei et al., 2021; Alam et al., 2022; Wang et al., 2022), triple-negative breast cancer (Alam et al., 2022), esophageal cancer (ESCA; Xu et al., 2021), hepatocellular carcinoma (Lin et al., 2008; Li and Xu, 2020; Yang et al., 2021; Hu et al., 2022; Li et al., 2022; Qiao et al., 2022; Tan et al., 2022; Hasan et al., 2023; Hossen et al., 2023), glioblastoma (Visnyei et al., 2011; Qin et al., 2023), epithelial ovarian cancer (Brüning-Richardson et al., 2011; Alsiary et al., 2014; Wu et al., 2022), osteosarcoma (Liu et al., 2021), endometrial carcinoma (Liu et al., 2020; Zhang et al., 2022), malignant pleural mesothelioma (Zhang et al., 2020), cervical squamous cell carcinoma (Wen et al., 2020), lung adenocarcinoma (Feng et al., 2021; Hou et al., 2022; Tang et al., 2022; Yin et al., 2022; Zhang et al., 2022), anaplastic thyroid carcinoma (Fang et al., 2023), cutaneous squamous cell carcinoma (Su et al., 2022), human sarcomas (Tu et al., 2022), pancreatic ductal adenocarcinoma (Shi et al., 2022), anaplastic thyroid cancer (Jiang et al., 2022), and diffuse large B-cell lymphoma (Wu et al., 2021). Furthermore, analysis of The Comparative Toxicogenomics Database revealed ASPM as a hub gene in adenoid cystic carcinoma (Liu et al., 2023) and mucinous gastric carcinoma (Li et al., 2023). Thus, a huge amount of evidence points to a positive correlation between ASPM and cancer. Nonetheless, despite the function of ASPM in cell division, mechanistic details relating to its role in tumorigenesis require further investigation. A deeper understanding of the connection between ASPM and cancers will be critical to aid diagnosis and facilitate the development of therapeutic targets for tumorigenesis.

**Table 2:** *ASPM* mutations in multiple cancers.

Type of mutation	Mutation site	Location (exon)	Predicted protein effect	Primary Histology (Histology subtype 1)	References
Coding silent	c.5961A > G, p.Q1987=	18	Substitution	Lymphoid neoplasm (diffuse large B cell lymphoma)	Morin et al. (2016)
Missense	c.10338G > C, p.K3446N	26	Substitution	Glioma (astrocytoma Grade IV)	
Coding silent	c.7185C > T, p.F2395=	18	Substitution	Carcinoma (adenocarcinoma)	Dong et al. (2018)
Missense	c.5656C > T, p.R1886C	18	Substitution	Carcinoma (basal cell carcinoma); malignant melanoma (NS); malignant melanoma (nodular); carcinoma (signet ring adenocarcinoma)	Sharpe et al. (2015), Bonilla et al. (2016), Hayward et al. (2017), Lau et al. (2018), Rabbie et al. (2021)
Missense	c.7939C > A, p.L2647I	18	Substitution	Lymphoid neoplasm (diffuse large B cell lymphoma); hematopoietic neoplasm (acute myeloid leukemia); carcinoma (adenocarcinoma)	Morin et al. (2016)
Frameshift	c.5149del, p.I1717*	18	Deletion	Carcinoma (adenocarcinoma); carcinoma (serous carcinoma);	Cancer Genome Atlas (2012), Jones et al. (2012), Liu et al. (2014), Mouradov et al. (2014), Chen et al. (2015), Gingras et al. (2016)
Missense	c.7684A > G, p.S2562G	18	Substitution	Carcinoma (adenocarcinoma); carcinoma (hepatocellular carcinoma); hematopoietic neoplasm (acute myeloid leukemia)	
Missense	c.4495C > T, p.R1499W	18	Substitution	Carcinoma (adenocarcinoma); carcinoma (endometrioid carcinoma); malignant melanoma (NS)	Mouradov et al. (2014), Xicola et al. (2018)
Nonsense	c.4732C > T, p.R1578*	18	Substitution	Carcinoma (endometrioid carcinoma); carcinoma (adenocarcinoma); malignant melanoma (NS); malignant melanoma (*in situ* melanotic neoplasm)	Rabbie et al. (2021)
Coding silent	c.7674C > T, p.I2558=	18	Substitution	Carcinoma (adenocarcinoma); carcinoma (hepatocellular carcinoma); hematopoietic neoplasm (acute myeloid leukemia)	
Missense	c.2929C > T, p.R977C	9	Substitution	Glioma (astrocytoma Grade IV); carcinoma (adenocarcinoma)	Giannakis et al. (2016), Nomura et al. (2017)
Coding silent	c.2307A > C, p.A769=	5	Substitution	Glioma (astrocytoma Grade IV)	
Missense	c.5639C > T, p.S1880F	18	Substitution	Carcinoma (basal cell carcinoma); malignant melanoma (NS)	Bonilla et al. (2016), Hayward et al. (2017)
Coding silent	c.4449A > G, p.K1483=	18	Substitution	Carcinoma (adenocarcinoma); carcinoma (hepatocellular carcinoma); hematopoietic neoplasm (acute myeloid leukemia)	
Missense	c.4213C > T, p.R1405C	17	Substitution	Lymphoid neoplasm (plasma cell myeloma); carcinoma (adenocarcinoma)	Giannakis et al. (2014), Giannakis et al. (2016), McMillan et al. (2018), Tessoulin et al. (2018)
Nonsense	c.9319C > T, p.R3107*	21	Substitution	Carcinoma (basal cell carcinoma); malignant melanoma (NS); carcinoma (endometrioid carcinoma)	Sharpe et al. (2015), Hayward et al. (2017)
Coding silent	c.3138G > A, p.R1046=	10	Substitution	Carcinoma (adenocarcinoma)	
Nonsense	c.9592C > T, p.R3198C	22	Substitution	Carcinoma (adenocarcinoma); malignant melanoma (NS)	Kumar et al. (2016)
Nonsense	c.6232C > T, p.R2078*	18	Substitution	Carcinoma (squamous cell carcinoma); carcinoma (endometrioid carcinoma)	Gao et al. (2014)
Missense	c.3155C > T, p.A1052V	10	Substitution	Carcinoma (adenocarcinoma); carcinoma (endometrioid carcinoma); malignant melanoma (NS)	Krauthammer et al. (2015), Giannakis et al. (2016)
Missense	c.2824C > T, p.R942C	8	Substitution	Carcinoma (adenocarcinoma); carcinoma (clear cell renal cell carcinoma); malignant melanoma (NS)	Giannakis et al. (2016), Hayward et al. (2017)
Missense	c.2822C > T, p.S941F	8	Substitution	Malignant melanoma (NS)	Sanborn et al. (2015)
Missense	c.4214G > A, p.R1405H	17	Substitution	Lymphoid neoplasm (acute lymphoblastic B cell leukemia); malignant melanoma (NS); carcinoma (squamous cell carcinoma)	Krauthammer et al. (2015), Hedberg et al. (2016), Li et al. (2017, 2020)
Missense	c.2752G > A, p.E918K	8	Substitution	Carcinoma (adenocarcinoma); carcinoma (endometrioid carcinoma); carcinoma (nasopharyngeal carcinoma)	Seshagiri et al. (2012), Liu et al. (2018)
Coding silent	c.1731C > T, p.S577=	3	Substitution	Carcinoma (adenocarcinoma); carcinoma (small cell carcinoma)	Peifer et al. (2012), Mouradov et al. (2014), George et al. (2015)
Frameshift	c.5039del, p.N1680Mfs*4	18	Deletion	Carcinoma (adenocarcinoma); carcinoma (NS)	Liu et al. (2014)
Nonsense	c.9730C > T, p.R3244*	23	Substitution	Glioma (astrocytoma grade IV); carcinoma (ductal carcinoma); malignant melanoma (NS)	Krauthammer et al. (2012)
Missense	c.1607A > G, p.K536R	3	Substitution	Adenoma (tubulovillous)	Saito et al. (2018)
Missense	c.5185C > T, p.R1729W	18	Substitution	Carcinoma (adenocarcinoma); Ccrcinoid-endocrine tumor (NS); malignant melanoma (NS)	Hintzsche et al. (2017), McMillan et al. (2018), Newell et al. (2019)
Nonsense	c.9454C > T, p.R3152*	21	Substitution	Glioma (astrocytoma grade IV); carcinoma (adenocarcinoma); carcinoma (endometrioid carcinoma)	
Missense	c.3463 T > G, p.Y1155D	13	Substitution	Carcinoma (adenocarcinoma); carcinoma (NS)	Abaan et al. (2013), Mouradov et al. (2014)
Missense	c.7598C > T, p.S2533F	18	Substitution	Carcinoma (squamous cell carcinoma); carcinoma (basal cell carcinoma)	Bonilla et al. (2016)
Nonsense	c.7324C > T, p.R2442*	18	Substitution	Carcinoma (adenocarcinoma); malignant melanoma (NS)	Giannakis et al. (2016), Hayward et al. (2017)
Missense	c.6978G > A, p.M2326I	18	Substitution	Carcinoma (squamous cell carcinoma); carcinoma (NS)	Zhang et al. (2015), Cheng et al. (2016)

NS = non-specific.

## 5. Conclusion

In this review, we provided a comprehensive overview of the pathogenic mechanisms underlying microcephaly and cancer caused by ASPM mutations. We highlighted the functional aspects of ASPM mutations in relation to the symmetric cell division, proliferation, differentiation, and self-renewal of neural stem/progenitor cells, as well as in genomic stability and disease pathogenesis. Loss or mutation of ASPM leads to abnormal mitotic events in *Drosophila*, mouse, ferret, and human cultured cells. This is likely due to the abnormal activity of the spindle assembly checkpoint or mitotic slippage. ASPM, together with several interacting partners, including MCPH proteins (CITK, MCPH2; Paramasivam et al., 2007; Gai et al., 2016; Jayaraman et al., 2016), katanin (Jiang et al., 2017), calmodulin (van der Voet et al., 2009), cyclin E (Capecchi and Pozner, 2015), FOXO (Paik et al., 2009), and UBE3A (Singhmar and Kumar, 2011), contributes to normal mitotic progression and neurogenesis. In addition to these functionally confirmed partners, numerous potential interactors have been identified in mass spectrometry data from NCBI database.^3^ These include proteins involved in DNA repair [TP53 (Liu et al., 2020), MTOR (Hein et al., 2015)], microtubule formation [Aurora A (Adhikari et al., 2020)], cell cycle regulation [CDC16 (Huttlin et al., 2017, 2021), CEP78 (Hein et al., 2015), MYC (Heidelberger et al., 2018)], transcription [CREB3 (Huttlin et al., 2021), FOXJ1 (Huttlin et al., 2017, 2021), T53INP1 (Huttlin et al., 2017)], protein degradation [CUL3 (Bennett et al., 2010; Kouranti et al., 2022), HERC2 (Galligan et al., 2015)], protein chaperoning [DNAJB7 (Huttlin et al., 2021), DNAJB8 (Huttlin et al., 2021)], apoptosis [MYC (Heidelberger et al., 2018)], cell proliferation [NPM1 (Fasci et al., 2018)], kinetochore organization [Ndc80 (Hutchins et al., 2010)], and ciliary motility [ODAD1 (Huttlin et al., 2021)]. This array of binding partners offers new insight into the potential functions of ASPM. However, elucidating the mechanisms underlying the cooperation of ASPM with these factors in microcephaly and other diseases will require further investigation.

Mouse models have been widely used to study the function of ASPM, and research has revealed that ASPM mutations are found not only in microcephaly but also in other diseases and disorders. Indeed, it has been discovered that mice with *Aspm* mutations also exhibit reductions in sperm count and motility, as well as major defects in the male and female germlines (Pulvers et al., 2010). These findings highlight the complexity of ASPM function. In addition to the mechanisms mentioned above, our group has uncovered potential mechanisms involving ASPM in DNA repair and the DNA replication response, thus advancing our understanding of ASPM from alternative perspectives (Xu et al., 2021; Wu et al., 2022).

Overall, the pathogenic mechanisms of microcephaly are complex, with more than 30 known disease-causing genes identified (Xu et al., 2020; Phan and Holland, 2021; Zaqout and Kaindl, 2021; Razuvaeva et al., 2023) and, as a result, an increasingly broad range of research directions. The analysis of such heterogeneous disorders will facilitate a better understanding of human brain development and evolution. Moreover, refining and revising our understanding of the significant contributions of ASPM to brain development and other diseases, including cancer, will provide guidance for the diagnosis and treatment of this rare heterogeneous disease.

## Author contributions

XX are responsible for the conception of this review and finalized the content of the manuscript. XW and ZL wrote the manuscript draft. XW, ZL, Z-QW, and XX scientifically edited the manuscript. All authors contributed to the article and approved the submitted version.

## Funding

This work was supported by the National Natural Science Foundation of China (NSFC); grants (32090031, 31761133012, and 31530016) and Shenzhen Science and Technology Innovation Commission projects grants (JCYJ20220818095616035 and JCYJ201805073000163).

## Conflict of interest

The authors declare that the research was conducted in the absence of any commercial or financial relationships that could be construed as a potential conflict of interest.

## Publisher’s note

All claims expressed in this article are solely those of the authors and do not necessarily represent those of their affiliated organizations, or those of the publisher, the editors and the reviewers. Any product that may be evaluated in this article, or claim that may be made by its manufacturer, is not guaranteed or endorsed by the publisher.

## Footnotes

^1^ http://gepia.cancer-pku.cn

^2^ https://cancer.sanger.ac.uk/cosmic

^3^ https://www.ncbi.nlm.nih.gov/gene/259266
